# Genomic architecture and functional effects of potential human inversion supergenes

**DOI:** 10.1098/rstb.2021.0209

**Published:** 2022-08-01

**Authors:** Elena Campoy, Marta Puig, Illya Yakymenko, Jon Lerga-Jaso, Mario Cáceres

**Affiliations:** ^1^ Institut de Biotecnologia i de Biomedicina, Universitat Autònoma de Barcelona, Bellaterra (Barcelona), Spain; ^2^ Departament de Genètica i de Microbiologia, Universitat Autònoma de Barcelona, Bellaterra (Barcelona), Spain; ^3^ ICREA, Barcelona, Spain

**Keywords:** inversions, supergenes, humans, phenotypic traits, gene expression

## Abstract

Supergenes are involved in adaptation in multiple organisms, but they are little known in humans. Genomic inversions are the most common mechanism of supergene generation and maintenance. Here, we review the information about two large inversions that are the best examples of potential human supergenes. In addition, we do an integrative analysis of the newest data to understand better their functional effects and underlying genetic changes. We have found that the highly divergent haplotypes of the 17q21.31 inversion of approximately 1.5 Mb have multiple phenotypic associations, with consistent effects in brain-related traits, red and white blood cells, lung function, male and female characteristics and disease risk. By combining gene expression and nucleotide variation data, we also analysed the molecular differences between haplotypes, including gene duplications, amino acid substitutions and regulatory changes, and identify *CRHR1, KANLS1* and *MAPT* as good candidates to be responsible for these phenotypes. The situation is more complex for the 8p23.1 inversion, where there is no clear genetic differentiation. However, the inversion is associated with several related phenotypes and gene expression differences that could be linked to haplotypes specific of one orientation. Our work, therefore, contributes to the characterization of both exceptional variants and illustrates the important role of inversions.

This article is part of the theme issue ‘Genomic architecture of supergenes: causes and evolutionary consequences’.

## 1. Human supergenes and inversions

Supergenes are defined as clusters of tightly linked functional genetic elements spanning hundreds of kilobases that control complex balanced phenotypes and are inherited as a unit owing to reduced or absent recombination within them [1–4]. Since recombination reduction is essential, the most common mechanism of supergene formation are inversions [2,4], in which single crossovers between heterozygotes lead to unbalanced gametes [5,6]. This allows the generation of highly divergent haplotypes accumulating a large number of sequence differences, which can form coadapted gene complexes [5,6]. In fact, there are many examples of inversion supergenes involved in adaptation in multiple organisms, such as the polymorphic social behaviour of ants, the Batesian mimicry wing colour pattering in butterflies or mating morphs in sparrows and ruffs, among many others [7–11].

In humans, there are practically no described cases of supergenes so far. Recent studies have finally started to characterize in detail human inversion polymorphism, but most inversions are relatively small and their potential functional effects are limited to nearby genes [12–14]. In addition, bigger inversions tend to be mediated by large complex segmental duplications (SDs), which could result in high rates of inversion recurrence by non-allelic homologous recombination (NAHR) that complicate finding phenotypic associations [13,14]. Two good human supergene candidates are the large inversions in 17q21.31, which has already been proposed to act as a supergene [15,16], and 8p23.1 [17,18]. The two inversions are well known and have been linked to diverse effects [12,19,20], although it has not been possible to establish clearly the actual genes and molecular changes responsible for them.

Here, we investigate to what extent the two inversions are indeed supergenes. By integrating the published information and newest genomic data, including nucleotide variation, gene expression and genome-wide association studies (GWAS), we give a global overview of their genomic architecture and how they can cause their effects. Also, we discuss briefly other potential human inversion supergene candidates.

## 2. The 17q21.31 inversion

**Figure 1.**
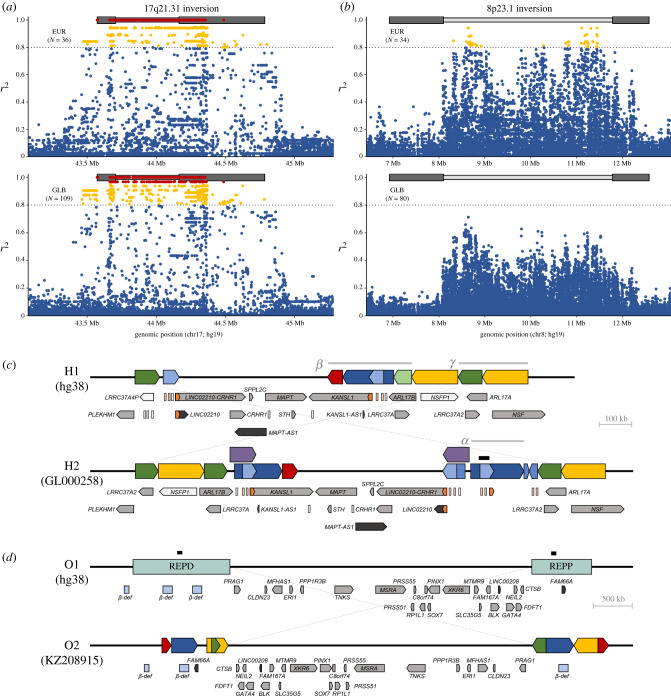
Summary of the genomic structure of two potential human inversion supergenes. (*a,b*) Linkage disequilibrium (LD) distribution of the 17q21.31 (hg38, chr17: 45 495 836–46 707 123) (*a*) and 8p23.1 (hg38, chr8:7 064 966–12 716 088) (*b*) inversions in European (EUR) and global (GLB) populations. Each dot represents a polymorphic variant within the inversion ± 500 kb region. LD was calculated from 1000 Genome Project (1000GP) individuals in which the inversion has been genotyped experimentally (*N*) and is shown in different colours: *r*^2^ < 0.8 (blue), *r*^2^ = 0.8–0.95 (yellow) and *r*^2^ > 0.95 (red). The rectangles above the graphs summarize the inversion region (light grey) and the variable segmental duplication (SD) blocks at its breakpoints (dark grey). (*c*) Structure of the inversion 17q21.31 H1 and H2 haplotypes updated from Boettger *et al*. and Steinberg *et al*. [21,22]. Coloured blocks indicate repeated segments at the breakpoints, with partial copies from the same repeat in lighter colours. The copy number variable fragments corresponding to *α*, *β* and *γ* duplications are indicated above [21]. Arrows below each orientation represent protein-coding genes (grey), non-coding genes (black) and pseudogenes (white). Small orange boxes correspond to the duplication of the first *KANSL1* exons and two pseudogenes. (*d*) Structure of the 8p23.1 inversion region according to Mohajeri *et al*. [24]. SD organization within the REPD and REPP repeat blocks in O1 (light green rectangles) is not indicated because it has not been completely resolved and it includes gaps (indicated as black bars) and known assembly errors. Owing to the size of the region, all protein-coding genes (grey arrows) and only two non-coding genes mentioned in the main text (black arrows) are shown, with β-defensin clusters pictured as light blue boxes.

The 17q21.31 inversion spans approximately 900 kb flanked by variable complex SD blocks of 200–800 kb and it is located within a 1.5-Mb long Chr. 17 region of high linkage disequilibrium (LD), defining two divergent haplotypes (H1 and H2) with no recombination between them [15,21,22]. The inversion was initially genotyped in a limited number of diverse individuals by fluorescence *in situ* hybridizaton (FISH) [22,23] and more recently by droplet digital polymerase chain reaction [14]. Thus, early on it was established that H1 and H2 correspond to the reference and inverted orientation, respectively, which results in many completely linked tag single nucleotide polymorphisms (SNPs) (figure 1*a*; electronic supplementary material, table S1).

Inversion imputation based on these tag SNPs showed a particular distribution pattern across the globe. H2 is low in Africa (less than 6%) and virtually absent in East Asian populations, while it forms a southwest/northeast cline in Europe, with highest frequencies in Mediterranean regions of southwest Asia and Europe (20–37%), high frequency in western, central, and southeast Europe (15–28%) and much lower frequency in eastern and northern Europe and on the Arabian Peninsula (5–10%) [22,25,26]. In addition, based on haplotype differentiation, the age of the inversion was estimated on 2–3 Myr [15,27] (although more recent estimates of approx. 100 000 years have been obtained using different methods [26]). According to primates, H2 is the ancestral orientation, although there could have been independent recurrent inversion events in human, chimpanzee and orangutan lineages [27]. Therefore, the derived H1 orientation probably increased in frequency to almost fixation in the human lineage, and a recent selective sweep of H2 haplotypes, possibly associated with higher fertility and recombination rate of H2 carrier females, resulted in the current distribution and lack of diversity of inverted chromosomes [15,27].

### (a) Inversion phenotypic effects

**Figure 2.**
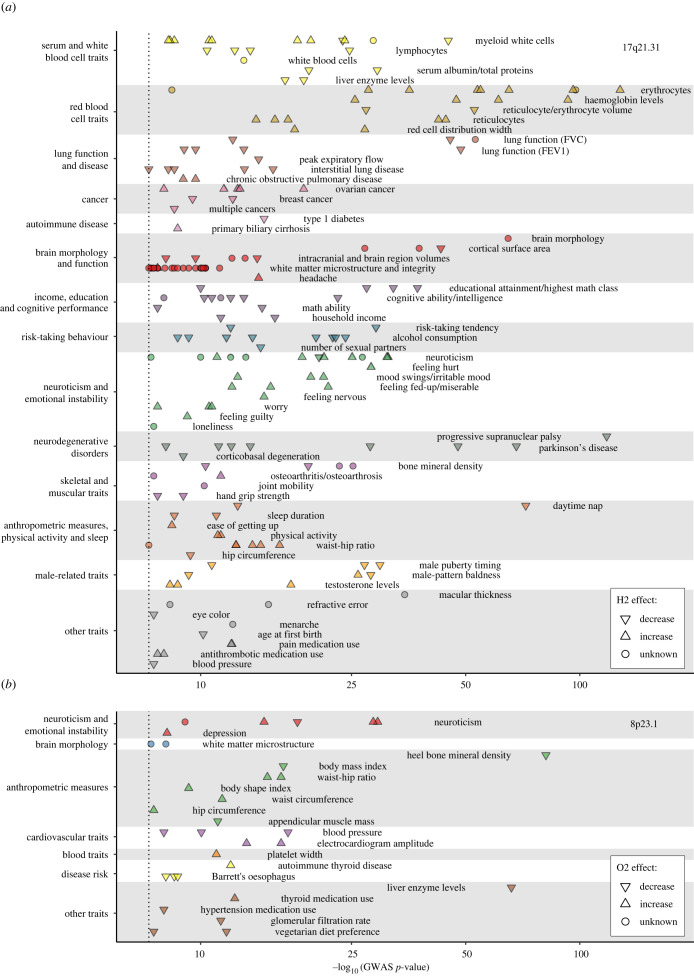
Summary of phenotypic associations for 17q21.31 (*a*) and 8p23.1 (*b*) inversions. GWAS Catalog signals (*p* < 5 × 10^–8^, dotted line) in high LD (*r*^2^ ≥ 0.8) with the inversions in the closest studied population are shown grouped by categories of related traits with different colours. Upwards and downwards triangles illustrate direction of effect of the inverted allele (H2 or O2) and dots unknown direction. Complete data are described in the electronic supplementary material, tables S2 and S3. (Online version in colour.)

The 17q21.31 inversion stands out for its pleiotropic phenotypic effects and it has been involved in an increasing number of diverse traits [12,14,19,20,28,29]. To update and understand better these effects, we have done an exhaustive analysis and manual curation of the GWAS hits (*p* < 5 × 10^−8^) corresponding to all SNPs in high LD (*r*^2^ > 0.8) with the inversion listed in the GWAS Catalog [30] (summarized together with their supporting studies in the electronic supplementary material, table S2). This has allowed us to classify the 203 independent hits in 14 broad categories comprising 64 more or less different traits, showing quite consistent and related effects for the two orientations (figure 2*a*).

As shown in figure 2*a*, one of the first phenotypes the inverted haplotype has been associated with is a lower risk of several neurodegenerative disorders, including Parkinson's disease, progressive supranuclear palsy and corticobasal degeneration [12,20]. However, the H2 haplotype is also associated with very significant changes in cell blood composition (electronic supplementary material, table S2). These changes combine higher levels of mature and immature erythrocytes, haemoglobin and haematocrit (together with more variable and smaller cell volume), and a general increase of white blood cells, especially neutrophils and basophils, but not eosinophils and lymphocytes, that show lower counts. In addition, the inversion is linked to a reduction of several lung function parameters and to increased risk of chronic obstructive pulmonary disease, although it could protect from lung fibrosis (electronic supplementary material, table S2). It is also linked to opposite effects in ovarian (higher risk) and breast cancer (lower risk), which might be related to the different role of progesterone in these cancers, and in autoimmune diseases like type 1 diabetes (lower risk) and primary biliary cirrhosis (higher risk) (electronic supplementary material, table S2).

A large group of related phenotypes are those involved in brain morphology and different cognitive and behavioural traits (figure 2*a*). For example, H2 is associated with reduced intracranial volume and cortical surface area, plus additional changes in white matter microstructure and integrity (electronic supplementary material, table S2). Actually, a genetic correlation has been found between reduced brain surface and several of the inversion-associated traits, such as higher tendency to neuroticism and depressive behaviours, and a decrease of the highly interrelated variables cognitive ability, education and household income [31,32]. Similarly, apart from reduced sleep, the inversion appears to be associated with higher physical activity and less inclination towards risk-taking behaviours, like speeding, alcohol consumption and number of sexual partners (electronic supplementary material, table S2).

Finally, H2 is associated with several anthropometric, skeletal and muscular parameters that could be connected with the previous phenotypes: lower blood pressure; higher waist-hip ratio, which contrasts with the increased activity levels and could be caused by smaller hip circumference; and reduced hand grip strength and bone mineral density, combined with higher risk of osteoarthritis (figure 2*a*; electronic supplementary material, table S2). The inversion is also associated with alopecia, later puberty and higher testosterone levels in males (electronic supplementary material, table S2), although there is not an obvious relationship between them. In females, as already mentioned, the inversion was originally linked to higher fertility and higher recombination rates [15,33], which is confirmed here by a reduction in the age at first birth plus an association with menarche onset (electronic supplementary material, table S2). Other inversion associations involve eye phenotypes, including lighter colour, typical of Europeans where the inversion is most common (figure 2*a*; electronic supplementary material, table S2). Interestingly, the observed increase in medication use fits well with some other possible inversion effects, such as antithrombotic medication and higher number of red blood cells and pain medication and higher risk of headache and arthritis (electronic supplementary material, table S2).

Therefore, the number and diversity of inversion-associated traits make it clearly the best supergene example in humans. Moreover, our analysis suggests that H2 selection in Europeans could be more complex than initially thought, involving other traits besides fertility. In particular, there are several phenotypes potentially involved in immune response, a highly selected trait, and it has been recently found that the inversion protects against severe COVID-19 [29], which could be related to changes in immune cells, lung function or even red blood cell levels. Nevertheless, so far there is little information about the molecular causes behind these phenotypic differences.

### (b) Sequence differences between inversion haplotypes

Possible functional effects of 2568 variants that differentiate H1 and H2 haplotypes across diverse human populations (*r*^2^ > 0.95) were analysed using the Ensembl Variant Effect Predictor (VEP) [34] (electronic supplementary material, table S4). We found 17 SNPs that cause amino acid (aa) changes in five genes, with *SPPL2C*, *MAPT* and *KANSL1* accumulating 7, 5 and 3 aa differences, respectively (table 1). Of those, two changes in *SPPL2C* and *KANSL1* and one in *MAPT* have combined annotation dependent depletion (CADD) scores greater than 20, indicating that they are among the 1% most deleterious substitutions in the human genome (electronic supplementary material, table S4). Most of these variants affect the proteins produced by several alternatively spliced isoforms of the genes. There are also two SNPs in positions adjacent to splicing signals in the non-coding RNA *LINC02210*, which could affect the splicing of four different transcripts (table 1). In addition, 60 SNPs are located within sequences annotated as promoters in the Ensembl Regulatory Build [35], affecting up to 12 promoters of nine different genes (table 1). For example, a putative promoter region of the shorter *KANSL1* transcripts and/or the *KANSL1-AS1* long non-coding RNA (lncRNA) (ENSR00000095038), which is highly conserved in vertebrates, includes four nucleotide changes with CADD scores greater than 17. Finally, we detected many other SNPs that could affect gene regulation by altering untranslated regions (UTRs) or other types of regulatory elements, like enhancers or CTCF-binding sites (table 1; electronic supplementary material, table S4).

**Table 1.** Potential functional effects of SNP differences in high LD (*r*^2^ > 0.95) with 17q21.31 H1 and H2 haplotypes according to VEP analysis. (The combined annotation dependent depletion (CADD) value scores the predicted deleteriousness of variants by integrating multiple annotations, including conservation and functional information. Variants with scores greater than 20 are predicted to be the 1% most deleterious substitutions in the human genome. n.a., not applicable.)

SNP effect	no. SNPs	affected elements	no. genes	genes
amino acid change (CADD > 20)	5	n.a.	3	*SPPL2C, MAPT, KANSL1*
amino acid change (CADD < 20)	12	n.a.	5	*CRHR1, SPPL2C, MAPT, STH, KANSL1*
synonymous change	16	n.a.	4	*CRHR1, SPPL2C, MAPT, KANSL1*
UTRs	58	n.a.	3	*CRHR1, MAPT, KANSL1*
splicing signals	2	n.a.	1	*LINC02210*
promoters	60	12	9	*DND1P1, RPS26P8, LINC02210, CRHR1, MAPT, KANSL1, KANSL1-AS1, DND1P2, RP11-359G18.1*
transcription factor binding sites	25	43	n.a.	n.a.
enhancers	76	24	n.a.	n.a.
CTCF binding sites	126	49	n.a.	n.a.

Apart from small sequence changes, the 17q21.31 inversion breakpoint regions (BP1 and BP2) are characterized by a considerable amount of structural diversity [21,22]. Within H1 chromosomes BP2, there are two copy number variable regions: duplication *β* (approx. 267 kb, 1–3 copies), containing two protein-coding genes (*ARL17A* and *LRRC37A2*), the first exons of *KANSL1*, *KANSL1-AS* and two pseudogenes (defective copies of *MAPK8IP1* and *DND1*); and duplication *γ* (approx. 218 kb, 1–4 copies), which includes *ARL17B* and *LRRC37A* and part of *NSF* (pseudogene *NSFP1*) (figure 1*c*). Both duplications were generated independently from a haplotype with a single copy of each sequence (H1.*β*1.*γ*1), with the most common H1 haplotypes in human populations being H1.*β*1.*γ*1, H1.*β*2.*γ*1, H1.*β*1.*γ*2 and H1.*β*1.*γ*3 [21,22].

The inversion originated probably in an H1.*β*1.*γ*2 haplotype by NAHR between 59 kb SDs containing copies of genes *ARL17* and *LRRC37* in opposite orientation (figure 1*c*) [27]. However, inverted chromosomes have at least 32 kb extra sequence that creates the approximately 85 kb H2-specific inverted SDs at both breakpoints (figure 1*c*). Moreover, the most common H2 haplotype (H2.*α*2.*γ*2) contains an additional insertion in BP2 of around 200 kb, formed probably by a complex rearrangement of a complete 154 kb *α* duplication and two 12.3- and 28.4 kb *α* fragments (although a gap in the only available sequence complicates the analysis) [21,22]. Other H2 haplotypes with a deletion of the *γ* duplication in BP1 also exist at low frequencies [21,22]. Therefore, H2.*α*2.*γ*2 gene content has three main differences from haplotype H1.*β*1.*γ*2: (i) two functional *LRRC37* copies (BP2) and one pseudogene (BP1) in H1 versus three functional copies in H2; (ii) an extra copy of the first three exons and an alternative first exon of *KANSL1* and their respective promoters, which could generate novel transcripts in H2; and (iii) up to four copies of *DND1* and *MAPK8IP1* pseudogenes in H2 versus only two copies of each in H1 (figure 1*c*).

Besides these large changes at the breakpoints, we looked at the LD of the inversion with other structural variants (SVs) identified in the 1000GP [36,37] (electronic supplementary material, table S5). A 1383 bp SVA element insertion and two deletions (323 and 314 bp long) in different *KANSL1* introns were associated with H1/H2 haplotypes (*r*^2^ > 0.8). In addition, a 238 bp *MAPT* intronic deletion that had been proposed to map to H2 haplotypes [15] showed a *r*^2^ = 0.85, which could be caused by errors in deletion genotype imputation.

### (c) 17q21.31 Haplotypes and gene expression

**Figure 3.**
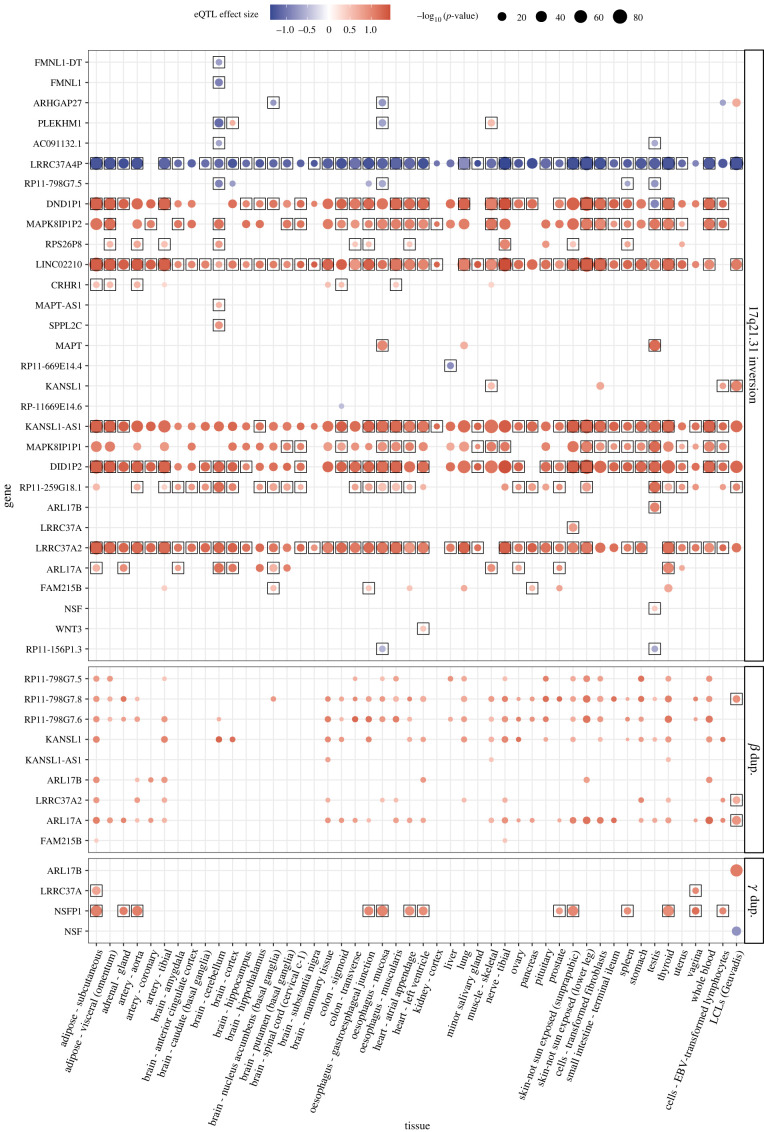
Tissue-specific association of gene expression with the inversion and *β* and *γ* duplications in the 17q21.31 region. Variant *cis*-eQTL effects were estimated by testing associations between genotypes and gene expression measures from multiple GTEx tissues and Geuvadis lymphoblastoid cell lines (LCLs) [38,39] (see the electronic supplementary material, methods). eQTL effect size and direction associated with the presence of the inversion or duplication is illustrated by the colour gradient and the *p*-value by the dot size, with black squares indicating when the variants are lead eQTLs or in high LD (*r*² ≥ 0.95) with top variants in the corresponding dataset (electronic supplementary material, table S6). Genes are ordered according to their genomic position. Figure is an updated version of that in Degenhardt *et al.* [29].

We identified gene expression differences associated with the 17q21.31 inversion and the *β* and *γ* duplications in multiple tissues and cell lines using Geuvadis and GTEx RNA-Seq data [38,39] (electronic supplementary material, table S6). Similar to previous results obtained from GTEx expression quantitative trait loci (eQTLs) information [14,29], the inversion acts as lead eQTL in at least one tissue of 29 genes located both within the inversion and in the nearby flanking regions (figure 3). Of those, 13 are protein-coding genes, nine non-coding RNAs and seven pseudogenes. Remarkably, the genes flanking BP1 tend to be downregulated in H2, whereas most of those within the inverted region and around BP2 are upregulated (figure 3). Actually, the inversion is associated with an alteration of *cis*-regulatory domains in the region, defined by correlated histone modifications, that mirrors the changes in expression patterns [40]. However, the consequences of this alteration are not known, and sequence differences between H1 and H2 could also be responsible. For example, in H2, missing pseudogene *LRRC37A4P* is consistently downregulated (figure 1*c*), whereas *LRRC37A2* is upregulated in almost all tissues (figure 3), which could be explained by the presence of three functional *LRRC37* copies. Similarly, the *DND1* and *MAPK8IP1* pseudogenes that have additional copies in H2 show widespread increased expression (which contrasts with the lack of expression differences associated with the H1 *β*-duplication, containing extra copies of these pseudogenes as well; see below).

With respect to tissue distribution, many changes in gene expression accumulate in different parts of the brain, especially in the cerebellum (figure 3), a structure linked to some of the symptoms of the neurodegenerative diseases against which the inversion has protective effects [12,20]. In fact, the genes in the 17q21.31 inversion region show highest expression in only a few tissues [29,39]: cerebellum, pituitary, lung, testis and female reproductive organs for protein-coding genes and non-coding RNAs; and testis, followed by cerebellum and female reproductive organs, for pseudogenes. Thus, expression differences associated with both haplotypes (figure 3) are expected to have more functional consequences in these tissues, which could explain the phenotypic changes related to them.

The *β* and *γ* duplications were associated with expression differences of nine and four genes, respectively (figure 3). In this case, lower imputation accuracies complicate assessing if they are the causal variant, but the duplications were the lead eQTLs of at least three (*β*) and two (*γ*) genes. Moreover, most of these genes are upregulated and map inside the duplicated segments. Exceptions are the partially duplicated *NSF* gene, which is downregulated in lymphoblastoid cell lines (LCLs) with duplication *γ*, and duplication *β* pseudogenes *MAPK8IP1P1* and *DND1P2*, which do not show higher expression with more *β* copies. Some of the genes that could be affected by the *β* and *γ* duplications also show expression changes between H1 and H2 haplotypes. However, only the *KANSL1* expression change can be explained by the *β* duplication, although the inversion has a much stronger effect in just certain tissues (figure 3). Therefore, the observed expression changes appear to be more associated with the two inversion haplotypes than with the presence of either duplication.

### (d) Candidate integrative analysis

As usually happens with supergenes, the huge number of variants in high or almost complete LD complicates narrowing down the individual genetic changes responsible for the different phenotypes. Nevertheless, based on the sequence and expression changes observed and functional information of the genes, there are several good candidates to mediate the 17q21.31 inversion phenotypic associations.

One of the best candidates is *CRHR1*, which encodes a receptor involved in corticotropin-releasing hormone (CRH) family signalling. This protein is a major regulator of the hypothalamic-pituitary-adrenal (HPA) axis and activates transduction pathways that regulate diverse physiological processes in which the inversion could have an effect, such as stress, the immune system, mood and emotions, reproduction, sleep and obesity [41]. Importantly, the CRHR1 receptor is also involved in promoting embryo implantation [42] and modulates myometrial contractility [41], which could be related to the inversion effect on fertility. Therefore, the inversion probably increases CRHR1 activity and HPA axis signalling. According to our analysis (figure 3), H2 is associated with upregulation of *CHRH1* in several tissues but not in brain, where the gene is most highly expressed (although a limited previous study using microarrays found increased *CHRH1* expression in the cerebellum [43]). However, the inversion is lead eQTL for *LINC02210* upregulation in almost all tissues. This lncRNA forms a fusion transcript with *CRHR1* and could be driving a higher expression of some *CRHR1* isoforms (figure 1*c*), which is consistent with changes in *LINC02210* splicing patterns between the two haplotypes in GTEx data [29].

Another good candidate is *KANSL1,* which produces a histone acetylation regulatory subunit that could be related to transcription regulation and chromatin modification. *KANSL1* pathogenic mutations in heterozygosis cause developmental psychomotor delay and mild/modest intellectual disability characteristic of Koolen-de Vries syndrome [44,45]. Moreover, the KANSL1 protein is involved in microtubule organization during mitosis [46]. The H2 haplotype is associated with higher *KANSL1* expression just in muscle and cultured LCLs and fibroblasts, whereas the overlapping *KANSL-AS1* transcript (figure 1*c*) is upregulated in all tissues (figure 3). These expression differences could be in part owing to the nucleotide changes accumulated in the joint promoter of the shorter *KANSL1* isoforms and *KANSL1-AS1*. In addition, different *KANSL1* coding and non-coding isoforms show inversion-associated expression changes in other conditions, such as Parkinson disease patients [47] or monocytes under immune stimuli [29], emphasizing the potential important functional consequences of this gene in many tissues and phenotypes.

The gene *MAPT* has been long implicated in neurodegenerative diseases caused by aggregation of the encoded microtubule-associated protein tau, which promotes assembly and stabilization of cytoskeletal microtubules [48]. It is expressed predominantly in the central nervous system, where there are six MAPT transcripts produced by alternative splicing of exons 2, 3 and 10 [49]. Alternative splicing of exon 10 results in MAPT protein isoforms with three (3R) or four (4R) repeat microtubule-interacting domains, and mutations disrupting the normal exon 10 splicing balance cause disease [49]. We have seen that *MAPT* expression changes are limited to H2 upregulation in the oesophagus, testis and lung (figure 3). Previous microarray expression data found increased *MAPT* expression in H1 chromosomes in frontal cortex and cerebellum [43,50], but it has been suggested that this difference was probably caused by a technical artefact [51]. However, reporter assays indicated that transcriptional activity of H1 *MAPT* promoter was higher than H2 [52]. In addition, several studies have reported *MAPT* splicing differences between H1/H2 haplotypes, such as higher expression of the isoform containing exon 10 in H1 [53,54] or the one containing exon 3 in H2 [51,55]. These differences are also supported by the association of the inversion with multiple splicing QTLs (sQTLs) in GTEx in several brain areas [29]. Remarkably, between H1 and H2 sequences, there are 42 SNPs plus the 238 bp deletion within the intron preceding exon 10 that could be affecting the splicing of this critical exon. Two additional *MAPT* SNPs linked to H1/H2 haplotypes produce aa substitutions with high CADD scores: rs17651549 (CADD = 26.9) and rs10445337 (CADD = 18.9). The most deleterious substitutes arginine (polar) by tryptophan (hydrophobic) in a position conserved in all mammals, although both changes occur in exons included in MAPT isoforms expressed in the peripheral nervous system and their consequences are not known [49]. Thus, the molecular mechanisms involved in neurodegenerative disease risk are not yet clear, but both *MAPT* increased expression and preferential inclusion of exon 10 could play a role.

One last candidate that accumulates many changes between H1 and H2 haplotypes is *SPPL2C*, which has seven aa substitutions with CADD scores ranging from 0.001 to 23.3. This gene encodes a signal peptide peptidase of 684 aa that is expressed predominantly in testis and could be related to male germ cell development and fertility [56], although it is upregulated in H2 in cerebellum (figure 3). In particular, H2 SPPL2c has a proline for arginine substitution in position 461 that might be important for protein conformation according to the predicted interaction with other positions. So, in summary, different type of molecular changes in diverse genes could contribute to the widespread phenotypic effects of the 17q21.31 inversion.

## 3. The 8p23.1 inversion

The other well-characterized human inversion is that in 8p23.1, which spans a 4.2 Mb region on Chr. 8 [17,18,24]. This inversion was mediated by NAHR between large blocks of high-identity SDs spanning 0.8–1.0 Mb, that are typically known as REPD and REPP [18,24,57] (figure 1*d*). The complexity of the breakpoints makes studying of the 8p23.1 inversion very challenging and the reference (O1) or inverted (O2) orientation status has been determined experimentally for just around 200 individuals by FISH [17,18,23]. This has shown that the genetic structure is very different from the 17q21.31 inversion, without two clearly differentiated haplotypes and relatively low LD with other variants in most populations (figure 1*b*). The only exception is Europeans, that have several highly linked SNPs close to each breakpoint (*r*^2^ = 0.94) (electronic supplementary material, table S1). Such LD pattern is consistent with double crossovers homogenizing the central region of the inversion, as happens in large *Drosophila* inversions, and a small degree of recurrence [18,20,23]. Actually, it has already been proposed that the inversion has occurred independently in humans and the chimpanzee-bonobo lineage from the ancestral O2 orientation [18,23].

Nevertheless, several computational algorithms based on genetic differentiation have made it possible to impute 8p23.1 inversion genotypes quite reliably, especially in European individuals [18,58,59]. Although these estimates have limitations, they have shown that the inversion follows a clinal distribution in which the geographical distance from Ethiopia correlates negatively with O2 frequency, with the highest frequency in African populations (60–80%), followed by Europeans (50–60%) and East Asians (10–20%) [18]. Therefore, 8p23.1 geographical distribution agrees with O2 ancestral status and human population expansion out of Africa, suggesting very weak or neutral selective pressure.

### (a) Inversion phenotypic effects

The imputation methods have also allowed linking the 8p23.1 inversion to diverse phenotypic effects, although much fewer than in 17q21.31. It is well known that the O1 orientation is associated with a higher risk for systemic lupus erythematosus (SLE) [18,60,61]. Moreover, the presence of the inversion has been associated with neuroticism [62] and several risky behaviour traits [63]. However, it has been related to a reduction of different metabolic risk factors, such as hypertension, obesity and the co-occurrence of diabetes, hypertension or asthma with obesity, and to an increase of triglyceride levels [28,58].

Our extended analysis of the most recent GWAS Catalog data with highly linked SNPs (*r*^2^ > 0.8) in Europeans (figure 2*b*) replicates several of these inversion associations, including increased risk of neuroticism and depression, together with brain microstructure changes, or protection against hypertension and changes in other cardiovascular traits, like platelet width and electrocardiogram. In the case of obesity, we also find a lower body mass index with the inversion, but this is accompanied by an increase in diverse anthropometric measures typically related to obesity, such as both waist and hip circumference, waist-hip ratio or body shape index, and a reduction in muscle mass and bone mineral density (electronic supplementary material, table S3). This suggests that the role of the inversion in obesity and body plan might be complex. In addition, we have found new interesting inversion phenotypic associations, like protection against Barrett's oesophagus inflammation, increased risk of autoimmune thyroid disease, and lower liver enzyme levels, glomerular filtration rate and vegetarian diet preference (electronic supplementary material, table S3). Nevertheless, owing to the low LD with SNPs in many human populations, the phenotype analysis is limited and many effects are probably missed. Thus, more studies that determine directly inversion orientation in different GWAS datasets are needed.

### (b) 8p23.1 Inversion sequence and expression differences

To determine the potential molecular mechanisms responsible for 8p23.1 inversion phenotypes, we also checked the associated sequence and gene expression changes. As mentioned above, this inversion does not have a clear haplotype structure with a large number of tag SNPs in high LD (figure 1*b*). In 1000GP Europeans, only nine SNPs have a *r*^2^ > 0.9 with the inversion and none of them affects the protein sequence or alters 5′ or 3′ UTRs (electronic supplementary material, table S4). CADD scores associated with these changes are all below 10 except for rs13255193 (CADD = 10.25). Furthermore, only rs560544 locates within a CTCF binding site and affects four putative transcription factor binding sites.

On the other hand, the exact structure of the complex SD blocks flanking the inversion is still not fully resolved [24,57]. These SD blocks contain large β-defensin gene clusters that show considerable variation between individuals [57]. A complete characterization of a 6.3 Mb alternate reference assembly of the inverted sequence has shown 20 structural differences ranging from 1 to 357 kb with respect to the non-inverted reference assembly, the largest of which is another polymorphic inversion [24]. Moreover, the amount of copy number variation in the breakpoint regions between humans could be as much as 1.2 Mb [24]. However, it has not yet been possible to do similar detailed analysis of the SVs in multiple individuals and their association with inversion status. Currently, there are not any known SVs from the 1000GP data [36,37] in high LD with the inversion, although this analysis probably misses most of the variation in the duplicated regions.

With regard to gene expression, previous studies have suggested that the 8p23.1 inversion influences the expression of more than 20 genes [18,28,62,64]. Apart from one spanning multiple tissues [28], most analysis were done in blood-derived cells, and consistent expression differences were found for *BLK, NEIL2, MSRA, CTSB, FDFT1, MFHAS1, MTMR9, FAM167A* and *PPP1R3B*. To test this in more detail, we imputed 8p23.1 inversion genotypes and analysed expression levels in Geuvadis LCLs [38] and GTEx tissues [39] (see the electronic supplementary material, methods). According to our strict criteria, the inversion is lead eQTL only of non-coding transcript *RP11-148O21.4* in cultured fibroblasts and is highly linked (*r*^2^ = 0.81) to the lead variant for *RP11-419I17.1* in the oesophagus (electronic supplementary material, table S6). Our analysis also confirmed the gene expression changes previously associated with the inversion, but other SNPs in lower LD were apparently the lead variant. Thus, as already suggested [18], the inversion regulatory effects could be primarily mediated by certain haplotypes specific of one orientation, although given the potential errors during imputation, the inversion could still be responsible of part of the expression variation.

### (c) Candidate integrative analysis

In this case, the lack of sequence differences between the two orientations probably limits the number of gene expression changes directly linked to the inversion, which together with its large gene content and the complexity of the breakpoint regions, make it difficult to identify the molecular mechanisms behind inversion phenotypic effects. In fact, variation in β-defensin content has been involved in different immune-related phenotypes, such as psoriasis and Crohn's disease [65], although how this variation is associated with the inversion has not been determined yet.

Aside from the β-defensins clusters, one of the main candidates for the phenotypic differences is *BLK*, which encodes a tyrosine kinase. Higher autoimmune disease risk in O1 orientation is apparently related to a common 16-kb O1 haplotype containing several risk alleles for SLE and rheumatoid arthritis [18,60,61]. These risk alleles are located in the bi-directional promoter region of *BLK* and *FAM167A* (figure 1*d*) and are associated, respectively, with decreased or increased expression of each gene [18,66]. The *BLK* overlapping transcript *RP11-148O21.4* also shows lower expression levels in SLE [67], and its upregulation in fibroblasts with O2 fits well with the protective effect of the inversion. Furthermore, there are independent SLE GWAS signals close to other genes showing expression differences between orientations, such as *CTSB*, *MFHAS1, PRAG1* and *CLDN23* [61], and SLE risk of this locus could be related to the action of many genes.

Another inversion phenotypic association that could involve *BLK* is Barrett's oesophagus [68]. Two variants in high LD with the inversion (*r*^2^ = 0.89) located in the promoter region and first intron of lncRNA *LINC00208*, just downstream of *BLK* (figure 1*d*), have been associated with O2 protection against Barrett's oesophagus [68]. These variants are also associated with downregulation of lncRNA *RP11-419I17.1* specifically in the oesophagus (electronic supplementary material, table S6). However, there is no more information about the possible consequences of this change. Similarly, it has been proposed that *BLK, FDFT1* and *FAM66A* could be involved in diabetes [28]. Finally, *PPP1R3B* has been linked with serum lipid levels [18], but in most analysis no significant expression changes between inversion orientations have been found.

## 4. Are there other human inversion supergenes?

In the last few years, extended accurate genotyping in multiple individuals of a larger number of inversions has shown that a few other of the bigger inversions affect diverse functional and phenotypic traits [13,14,69]. An example is HsInv0786, a 171 kb inversion on Chr. 16, which has been associated with different phenotypes, including obesity and co-occurrence of obesity with asthma or hypertension, intelligence, type 1 diabetes, red blood cell count and pediatric autoimmune diseases [14,28,70]. This inversion has also been involved in expression changes of several genes, such as upregulation of *SULT1A2* or *TUFM*, an elongation factor required for mitochondrial protein synthesis and potentially involved in energy metabolism [14,28,70]. However, in most cases, the relatively low LD with flanking SNPs precludes determining reliably the effects of these inversions in available GWAS and functional datasets, as happens for HsInv0290, a low-frequency Chr. 7 inversion of approximately 0.74 Mb [14]. Therefore, future large-scale genotyping of these and other inversions will help us understand better the role of inversion supergenes in the human genome. Interestingly, despite the added difficulty of recurrence, the lack of complete association between inversions and SNPs gives the opportunity to solve the long-debated question if their effects are mainly caused by the inversion itself or the combination of SNPs contained within [16].

Another important question is how inversion supergenes are maintained, given their expected negative fertility costs. Accordingly, we should assume that these costs were outweighed by some type of favourable selection. Thus, it is not surprising that common large inversions accumulate many different effects that probably were selected at some point during human evolution, although now they may increase our disease risk, as could be the case for obesity or autoimmune diseases.

## Data Availability

The data are provided in the electronic supplementary material [71].
